# Rise in intraocular pressure with elevator travel in post-vitrectomy patients

**DOI:** 10.1038/s41598-023-40416-x

**Published:** 2023-08-28

**Authors:** Posey P. Y. Wong, Nicole C. Tsim, Karen K. W. Chan, Ivan H. W. Lau, Andrew C. Y. Mak, Guy L. J. Chen, Lawrence P. L. Iu, Mary Ho, Alvin L. Young, Mårten Brelén

**Affiliations:** 1 Department of Ophthalmology and Visual Sciences, Prince of Wales Hospital, 30-32 Ngan Shing Street, Shatin, Hong Kong; 2 Department of Ophthalmology and Visual Sciences, The Chinese University of Hong Kong, 4/F Hong Kong Eye Hospital, 147K Argyle Street, Kowloon, Hong Kong

**Keywords:** Medical research, Retinal diseases

## Abstract

To evaluate the impact of elevator travel on intraocular pressure after vitreoretinal surgery with gas tamponade. Patients undergoing pars plana vitreoretinal surgery with and without gas insertion were recruited on post-operative day 1. All intraocular pressures were measured three times by Tono-Pen AVIA (Reichert, USA) on the fourth floor and, after rapid ascent in an elevator, on the 12th floor of the hospital. All patients were observed and asked for any symptoms of pain or nausea for at least 15 min. In this study, 54 patients were recruited. Twenty-seven patients underwent vitreoretinal procedures with gas insertion, while 27 patients without gas insertion acted as controls. The mean age of patients was 60.9 years. The mean changes in intraocular pressure of the patients with gas insertion (+ 1.39 mmHg) were greater than those without gas insertion (− 0.43 mmHg) and statistically significantly different (95% CI 1.17–2.48, *P* < 0.0001). Patients undergoing vitreoretinal surgery with gas insertion had statistically significant intraocular pressure rise even with 8-floor ascent in the immediate post-operative period. Further studies are needed to evaluate the change in intraocular pressure with a larger range of altitudes and different gases.

## Introduction

Hong Kong ranks first in the world in terms of the number of skyscrapers, with at least 78 completed skyscrapers over the height of 200 m (656 ft), 517 skyscrapers over 150 m (492 ft), as well as more than 9000 high-rise buildings over 23 m (75 ft)^1,2^. The number of tall buildings in modern cities will inevitably increase given the global population rise and increased urbanisation.

Injection of intraocular gas is an integral part of many vitreoretinal surgeries. Patients are advised to avoid air travel as the low cabin pressures at high altitudes can cause expansion of the intraocular gas and induce dangerous elevations in intraocular pressure^3^. There is well-established evidence against air travel in post-vitrectomised patients with gas insertion. However, evidence on the effect of acute ascent in elevators in tall buildings on intraocular pressures in post-vitrectomised eyes with gas tamponade is still lacking.

The aim of this study was to evaluate whether travelling in elevators has any significant effect on intraocular pressure, and hence whether travelling in modern elevators in high-rise buildings should be avoided in the immediate postoperative period for patients with intraocular gas in-situ.

## Methods

This prospective case–control study was conducted according to the tenets of the Declaration of Helsinki and the study protocol was approved by the Joint Chinese University of Hong Kong—New Territories East Cluster Institutional Review Board (IRB). Written informed consent was obtained from all participants.

### Subjects

The inclusion criteria were patients undergoing pars plana vitreoretinal surgery with insertion of gas (Group A) and without gas insertion (Group B). Exclusion criteria were eyes with glaucoma, optic nerve atrophy, anti-glaucomatous medications. Pregnant or lactating women, children under 13 years old, patients unable to sit up independently or inability to give consent were also excluded.

### Data acquisition and analysis

Post-vitrectomy patients were recruited on post-operative day 1 at the Department of Ophthalmology and Visual Sciences at the Prince of Wales Hospital (PWH), Hong Kong between January 2016 and December 2021. Dilated fundal examination was done for each patient by vitreoretinal specialists who used standard clinical assessment techniques to determine the percentage of gas fill. All patients’ intraocular pressures were measured at the fourth floor and the 12th floor in the hospital 3 times by Tono-Pen AVIA (Reichert, USA). The mean value of the three measurements were recorded. The patients would be observed and asked for any pain and nausea after travelling to the 12th floor for 15 min.

### Statistical analysis

Statistical analysis was performed using the SPSS program (SPSS Statistics, version 26.0 for MacOS, SPSS Inc., IBM, Somers, NY). Independent sample T-test was used to determine any significant difference between the two groups. A *P*-value of less than 0.05 was considered statistically significant.

## Results

Fifty-four patients were recruited in our study. The mean age of all participants was 60.9 years. The number of male and female participants was 29 and 25 respectively. The gas used in 92% of patients with Group A was 12% C3F8. The demographic data and clinical features of the two groups of patients are summarized in Table 1. The percentage of patients with sclerotomy wound sutures in Group A and Group B was 88.89% and 37.03% respectively. This difference is attributed to a greater proportion of patients in Group A undergoing 23G pars plana vitrectomy (74.07%) and the majority of patients in Group B undergoing 25G vitrectomy (92.59%).

**Table 1:** Demographics and clinical characteristics of patients with (Group A) and without gas insertion (Group B) respectively.

	Number (%)
**Patients with gas insertion (Group A) 27 patients in total**	
**Mean age**	56.3
**Male**	12 (44.44%)
**Female**	15 (55.56%)
**Surgery**	
- RRD	20 (74.01%)
- RD	4 (14.81%)
- MH	2 (7.41%)
- VH	1 (3.70%)
**Gas inserted**	
- 12%C3F8	25 (92.59%)
- 20% SF6	1 (3.70%)
- Air	1 (3.70%)
**Percentage of gas filled**	
- 100%	9 (33.33%)
- 90%	12 (44.44%)
- 70%	4 (14.81%)
- 50%	2 (7.41%)
**Port size**	
- 23G	20 (74.07%)
- 25G	7 (25.93%)
** With sclerotomy wound sutures**	24 (88.89%)
**Adverse events after ascending in elevator**	
- Pain	1 (3.70%)
- Nausea	1 (3.70%)
**Patients without gas insertion (Group B) 27 patients in total**	
**Mean age**	65.2
**Male**	17 (62.96%)
**Female**	10 (37.04%)
**Surgery**	
- Epiretinal membrane peel	19 (70.37%)
- VH	6 (22.22%)
- Removal of silicone oil	1 (3.70%)
**Port size**	
- 23G	1 (3.70%)
- 25G	25 (92.59%)
- 27G	1 (3.70%)
**With sclerotomy wound sutures**	10 (37.03%)

*RRD* rhematogenous retinal detachment, *TRD* tractional retinal detachment, *MH* macula hole, *VH* vitreous haemorrhage.

The mean change of intraocular pressure of patients in Group A (with gas insertion) was + 1.39 mmHg (SD = 1.42) while that of Group B (without gas insertion) was − 0.43 mmHg (SD = 0.92). The range of mean intraocular pressure change of Group A and Group B were − 1–4.333 mmHg and − 2.333–1.333 mmHg respectively. The mean change in intraocular pressure of the patients with gas insertion was statistically significantly greater than those without gas insertion (1.83 mmHg, 95% CI 1.17–2.48, *P* < 0.0001). One patient with gas insertion felt nauseated after going up in the elevator. (Table 2).

**Table 2:** Mean intraocular pressure changes at different altitudes in patients with (Group A) and without gas insertion (Group B) respectively.

	Mean IOP at lower altitude (Range) in mmHg	Mean IOP at higher altitude (Range) in mmHg	Mean difference (mmHg) (SD)
Patients with gas insertion (Group A)	16.556 (8–24.333)	17.951 (12–25.667)	1.3951 (1.423)
Patients without gas insertion (Group B)	13.432 (6.667–21.667)	13.000 (6–22.333)	− 0.4321 (0.924)

*IOP* intraocular pressure, *SD* standard deviation.

## Discussion

Currently there is a lack of evidence to advise against travelling in tall skyscrapers for post-vitrectomised patients with gas insertion in the immediate post-operative period. In our locality, as in many other metropolitan cities, patients live in high-rise buildings, where in the postoperative period, they would normally travel by elevators to reach their home.

As the atmospheric pressure decreases with the ascent in altitude, an intraocular gas bubble will undergo expansion following Boyle’s law: P_1_V_1_ = P_2_V_2_, where P indicates the pressure of the system and V indicates the volume of the gas. The eye has several compensatory mechanisms including limited choroidal flattening, scleral expansion, and increased aqueous outflow, but these mechanisms are limited in their ability to accommodate expansion of the intraocular gas bubble^4^. Once the globe’s maximum capacity is reached, the intraocular pressure increases, which may result in acute glaucoma and even central retinal artery occlusion^5^.

In studies where the hypobaric chamber is used to simulate air travel, rises in intraocular pressure are seen with an average of 13 ± 3 mmHg to a peak of 26 ± 9 mmHg at 8000 feet in patients who have small intraocular gas bubble after scleral buckling surgery^6^. On the other hand, patients with a complete intraocular gas fill after pars plana vitrectomy who travelled by land through mountains with a peak ascent of 3895 ft, at a mean rate of 29 ft/min, had no adverse consequences. These patients had no retinal vascular occlusion, acute elevations of intraocular pressure, or symptomatic visual field loss attributable to elevated intraocular pressure^7^. Foulsham et al. presented a case report of a post-vitrectomy patient, 1 month after the procedure, who experienced a substantial increase in intraocular pressure from 14 to 41 mmHg. This increase occurred during a 14-min helicopter ascent of 2600 ft, with the patient's eye containing a 50% fill of 16% C_3_F_8_ gas. Although no significant adverse effects were reported, slow ascent rates with partially filled gas may also result in marked increases in intraocular pressure^8^. These studies highlight the importance that the rate of ascent has on changes in intraocular pressure in human eyes. As a result, it is well-known for post-vitrectomy patients with gas insertion to avoid air travel. Our pilot study provides insight into the change in intraocular pressure with a rapid ascent in elevators.

In this study, we utilized the Tono-Pen AVIA (Reichert, USA) to measure intraocular pressure due to its portability, independence from gravity, and minimal influence from corneal thickness^9^. The Tono-Pen has been shown to have a good correlation with the Goldmann applanation tonometer within a certain range of intraocular pressures (between 10 and 24 mmHg)^10^. In a study by Osman et al., the margin of intraocular pressure measurement in the normal patient group was reported to be ± 3.8 mmHg by the Tono-Pen XL and ± 2.7 mmHg by the Goldmann applanation tonometer^11^. We aimed to investigate the difference in intraocular pressure before and after elevator travel of the same eye of the same patient, using the same operator, thus minimizing the effect of measurement error. It should be noted that the Tono-Pen may underestimate intraocular pressure by 4.5 mmHg at an actual intraocular pressure of 15 mmHg in an air-filled eye, and by even more at higher intraocular pressures^12^. Therefore, it is plausible to speculate that the actual difference in intraocular pressure observed in our study could be even more significant^10,12^.

This study was limited by the number of floors in our hospital, and it is not possible to extrapolate our result to higher floors since atmospheric pressure decreases exponentially with altitude. Despite this limitation, a statistically significant change in intraocular pressure was found between the two study groups. In Hong Kong, most residential buildings are at least 30 stories and can reach 60–70 floors. The intraocular pressure changes on those patients living on upper floors is likely to be clinically significant. The study was also not standardized as there were different surgeons, port sizes, percentages of gas fill and suturing of sclerotomy wounds. Despite these limitations, the patients in both groups had good wound sealing without any leakage on post-operative day 1. Further standardized studies are needed to test a larger range of ascents to higher altitudes to establish guidelines on how high and how fast patients can ascend in elevators after vitreoretinal surgery. Future work could explore the use of mathematical modeling to simulate intraocular pressure changes at different altitudes (Supplementary Table S1).

In conclusion, the findings of this study indicate a statistically significant elevation in intraocular pressure among post-vitrectomy patients with gas insertion during elevator travel, even when traveling between a modest number of floors. As urbanization and population growth continue, this pilot study provides valuable insights and highlights the need for further investigation into the potential safety implications of elevated intraocular pressure in individuals residing in cities with abundant tall buildings and skyscrapers.

### Supplementary Information

Supplementary Table S1.

## Data Availability

The datasets generated during and/or analysed during the current study are available from the corresponding author on reasonable request.

## Abbreviations

CI: Confidence interval
SD: Standard deviation

## References

[1] Craighead G (2009). High-Rise Building Definition, Development, and Use.

[2] Emporis Skyline Ranking. (Emporis.com, 2015).

[3] Muzychuk AK, Adatia FA, Ford BA, Kherani AM (2011). Commercial air travel with a small intravitreous gas bubble. Arch. Ophthalmol..

[4] Lincoff H, Weinberger D, Stergiu P (1989). Air travel with intraocular gas II. Clinical considerations. Arch. Ophthalmol..

[5] Mills MD (2001). An assessment of intraocular pressure rise in patients with gas-filled eyes during simulated air flight. Ophthalmology.

[6] Noble J, Kanchanaranya N, Devenyi RG, Lam WC (2014). Evaluating the safety of air travel for patients with scleral buckles and small volumes of intraocular gas. Br. J. Ophthalmol..

[7] Levasseur SD, Rahhal FM (2013). Travel to high mountain elevations following vitrectomy with intraocular gas. Retina.

[8] Foulsham W, Chen XN, Vavvas DG (2021). Altitude-associated intraocular pressure changes in a gas-filled eye. Retin. Cases Brief. Rep..

[9] Bhan A (2002). Effect of corneal thickness on intraocular pressure measurements with the pneumotonometer, Goldmann applanation tonometer, and Tono-Pen. Invest Ophthalmol Vis. Sci..

[10] Wong B (2018). Comparison of disposable Goldmann applanation tonometer, ICare ic100, and tonopen XL to standards of care Goldmann nondisposable applanation tonometer for measuring intraocular pressure. J. Glaucoma.

[11] Osman EA, Gikandi PW, Al-Jasser AA, Alotaibi M, Mousa A (2018). Comparison of Goldmann applanation, noncontact air puff, and Tono-pen XL tonometry in normal controls versus glaucoma patients at a university hospital in Riyadh, Saudi Arabia. Middle East Afr. J. Ophthalmol..

[12] Kocak I, Koc H, Sayin N, Aydin A (2022). Tono-pen and Schiotz tonometer measurements in gas-filled eyes. J. Fr. Ophtalmol..

