# Supplementary material for: Rise in intraocular pressure with elevator travel in post-vitrectomy patients
Source: Sci Rep. 2023 Aug 28;13:14088. doi: 10.1038/s41598-023-40416-x (PMC10462696; doi:10.1038/s41598-023-40416-x)
Supplement: Supplementary file 1 — Supplementary Table S1. [file 41598_2023_40416_MOESM1_ESM.docx]

**Rise in Intraocular Pressure with Elevator Travel in Post-Vitrectomy Patients**

Supplementary Table

| Study No. | Age | Sex | Diagnosis | Eye | Gas | pIOP 1 | pIOP 2 | pIOL3 | Mean pIOP | IOP 1 | IOP 2 | IOP 3 | Mean IOP | Difference  mean  IOP-mean pIOP | Pain (Y/N) | Discomfort (Y/N) | % gas inserted | Port size (G) |
| --- | --- | --- | --- | --- | --- | --- | --- | --- | --- | --- | --- | --- | --- | --- | --- | --- | --- | --- |
| Group A | |  |  |  |  |  |  |  |  |  |  |  |  |  |  |  |  |  |
| 1 | 46 | M | TRD | RE | 12% C_3_F_8_ | 13 | 13 | 13 | 13 | 17 | 16 | 17 | 16.667 | 3.667 | N | N | 100 | 23 |
| 2 | 69 | F | MH RD | RE | 12% C_3_F_8_ | 20 | 25 | 26 | 23.667 | 21 | 27 | 27 | 25 | 1.333 | N | N | 100 | 23 |
| 3 | 54 | F | MH | LE | 12% C_3_F_8_ | 10 | 9 | 11 | 10 | 15 | 15 | 12 | 14 | 4 | N | N | 50 | 23 |
| 4 | 58 | M | TRD | LE | 12% C_3_F_8_ | 8 | 8 | 8 | 8 | 12 | 13 | 11 | 12 | 4 | N | N | 90 | 23 |
| 5 | 59 | F | TRD | LE | 12% C_3_F_8_ | 18 | 19 | 17 | 18 | 17 | 17 | 17 | 17 | -1 | Y | Y | 50 | 23 |
| 6 | 57 | M | Mac-off RD | RE | 12% C_3_F_8_ | 25 | 24 | 24 | 24.333 | 25 | 26 | 26 | 25.667 | 1.333 | N | N | 70 | 23 |
| 7 | 44 | F | Mac-off RD | RE | 12% C_3_F_8_ | 13 | 13 | 13 | 13 | 14 | 15 | 15 | 14.667 | 1.667 | N | Y (nausea) | 90 | 23 |
| 8 | 46 | M | Mac-on RD | LE | 12% C_3_F_8_ | 16 | 16 | 15 | 15.667 | 21 | 17 | 16 | 18 | 2.333 | N | N | 90 | 23 |
| 9 | 69 | F | TRD | LE | 12% C_3_F_8_ | 11 | 11 | 10 | 10.667 | 13 | 13 | 12 | 12.667 | 2 | N | N | 100 | 23 |
| 10 | 66 | F | Mac-on RD | LE | 12% C_3_F_8_ | 15 | 15 | 16 | 15.333 | 17 | 17 | 16 | 16.667 | 1.333 | N | N | 70 | 23 |
| 11 | 60 | F | Mac-off RD | RE | 12% C_3_F_8_ | 11 | 12 | 13 | 12 | 12 | 12 | 12 | 12 | 0 | N | N | 90 | 23 |
| 12 | 37 | M | Mac-off RD | RE | 20% SF_6_ | 17 | 19 | 19 | 18.333 | 21 | 21 | 21 | 21 | 2.667 | N | N | 90 | 23 |
| 13 | 40 | M | Mac-off RD | RE | 12% C_3_F_8_ | 19 | 18 | 19 | 18.667 | 20 | 20 | 20 | 20 | 1.333 | N | N | 70 | 23 |
| 14 | 67 | F | MH RD | RE | 12% C_3_F_8_ | 20 | 20 | 21 | 20.333 | 19 | 20 | 19 | 19.333 | -1 | N | N | 90 | 23 |
| 15 | 53 | M | Mac-on RD | RE | 12% C_3_F_8_ | 12 | 14 | 14 | 13.333 | 14 | 14 | 15 | 14.333 | 1 | N | N | 90 | 23 |
| 16 | 53 | M | Mac-off RD | RE | 12% C_3_F_8_ | 18 | 17 | 18 | 17.667 | 19 | 18 | 17 | 18 | 0.333 | N | N | 90 | 25 |
| 17 | 50 | M | Mac-off RD | RE | 12% C_3_F_8_ | 20 | 22 | 20 | 20.667 | 20 | 21 | 21 | 20.667 | 0 | N | N | 90 | 25 |
| 18 | 60 | F | Mac-off RD | RE | 12% C_3_F_8_ | 21 | 22 | 23 | 22 | 24 | 22 | 23 | 23 | 1 | N | N | 90 | 25 |
| 19 | 60 | M | Mac-off RD | RE | 12% C_3_F_8_ | 17 | 17 | 17 | 17 | 21 | 21 | 22 | 21.333 | 4.333 | N | N | 100 | 23 |
| 20 | 60 | F | MH RD | RE | 12% C_3_F_8_ | 15 | 15 | 15 | 15 | 15 | 15 | 15 | 15 | 0 | N | N | 100 | 25 |
| 21 | 68 | M | Mac-off RD | LE | 12% C_3_F_8_ | 21 | 22 | 22 | 21.667 | 21 | 22 | 22 | 21.667 | 0 | N | N | 100 | 25 |
| 22 | 44 | M | Mac-on RD | LE | 12% C_3_F_8_ | 15 | 16 | 17 | 16 | 17 | 17 | 18 | 17.333 | 1.333 | N | N | 90 | 23 |
| 23 | 54 | F | Mac-off RD | RE | 12% C_3_F_8_ | 11 | 12 | 12 | 11.667 | 13 | 14 | 14 | 13.667 | 2 | N | N | 90 | 25 |
| 24 | 64 | F | Mac-off RD | LE | 12% C_3_F_8_ | 16 | 17 | 17 | 16.667 | 18 | 18 | 19 | 18.333 | 1.667 | N | N | 100 | 25 |
| 25 | 57 | F | Mac-off RD | RE | 12% C_3_F_8_ | 20 | 21 | 22 | 21 | 21 | 22 | 22 | 21.667 | 0.667 | N | N | 100 | 23 |
| 26 | 66 | F | MH | RE | 12% C_3_F_8_ | 16 | 16 | 16 | 16 | 16 | 17 | 17 | 16.667 | 0.667 | N | N | 100 | 23 |
| 27 | 58 | F | VH | RE | Air | 17 | 17 | 18 | 17.333 | 18 | 18 | 19 | 18.333 | 1 | N | N | 70 | 23 |
|  |  |  |  |  |  |  |  |  |  |  |  |  |  |  |  |  |  |  |
| Group B | |  |  |  |  |  |  |  |  |  |  |  |  |  |  |  |  |  |
| 28 | 36 | M | VH | RE | Nil | 8 | 7 | 7 | 7.333 | 7 | 7 | 8 | 7.333 | 0 | N | N | 0 | 23 |
| 29 | 50 | M | VH + RD | LE | SO | 21 | 21 | 21 | 21 | 22 | 22 | 23 | 22.333 | 1.333 | N | N | 0 | 25 |
| 30 | 34 | F | ROO | RE | Nil | 13 | 13 | 14 | 13.333 | 14 | 13 | 14 | 13.667 | 0.333 | N | N | 0 | 25 |
| 31 | 68 | M | VH | RE | Nil | 22 | 22 | 21 | 21.667 | 22 | 21 | 21 | 21.333 | -0.333 | N | N | 0 | 25 |
| 32 | 70 | M | VH | LE | Nil | 7 | 7 | 7 | 7 | 7 | 8 | 7 | 7.333 | 0.3333 | N | N | 0 | 25 |
| 33 | 75 | M | ERM | LE | Nil | 14 | 15 | 15 | 14.667 | 13 | 12 | 14 | 13 | -1.667 | N | N | 0 | 25 |
| 34 | 74 | M | ERM | LE | Nil | 13 | 11 | 11 | 11.667 | 13 | 12 | 11 | 12 | 0.333 | N | N | 0 | 25 |
| 35 | 65 | F | ERM | LE | Nil | 8 | 8 | 8 | 8 | 6 | 9 | 7 | 7.333 | -0.667 | N | N | 0 | 25 |
| 36 | 71 | M | ERM | RE | Nil | 17 | 18 | 16 | 17 | 16 | 17 | 17 | 16.667 | -0.333 | N | N | 0 | 25 |
| 37 | 68 | F | ERM | LE | Nil | 9 | 10 | 9 | 9.333 | 10 | 10 | 10 | 10 | 0.667 | N | N | 0 | 27 |
| 38 | 48 | M | VH | LE | Nil | 20 | 18 | 18 | 18.6667 | 20 | 18 | 19 | 19 | 0.333 | N | N | 0 | 25 |
| 39 | 75 | M | ERM | LE | Nil | 12 | 14 | 14 | 13.333 | 12 | 11 | 11 | 11.333 | -2 | N | N | 0 | 25 |
| 40 | 72 | F | ERM | RE | Nil | 11 | 13 | 13 | 12.333 | 13 | 11 | 12 | 12 | -0.333 | N | N | 0 | 25 |
| 41 | 39 | M | VH | LE | Nil | 15 | 14 | 15 | 14.667 | 16 | 14 | 14 | 14.667 | 0 | N | N | 0 | 25 |
| 42 | 68 | F | ERM | RE | Nil | 14 | 12 | 13 | 13 | 15 | 13 | 13 | 13.667 | 0.667 | N | N | 0 | 25 |
| 43 | 75 | M | ERM | LE | Nil | 10 | 10 | 11 | 10.333 | 11 | 10 | 9 | 10 | -0.333 | N | N | 0 | 25 |
| 44 | 61 | F | ERM | LE | Nil | 7 | 6 | 6 | 6.333 | 6 | 7 | 5 | 6 | -0.333 | N | N | 0 | 25 |
| 45 | 60 | M | VH | RE | Nil | 12 | 11 | 10 | 11 | 12 | 11 | 11 | 11.333 | 0.3333 | N | N | 0 | 25 |
| 46 | 75 | M | ERM | LE | Nil | 11 | 10 | 12 | 11 | 10 | 10 | 11 | 10.333 | -0.667 | N | N | 0 | 25 |
| 47 | 81 | F | ERM | RE | Nil | 12 | 12 | 12 | 12 | 12 | 11 | 12 | 11.667 | -0.333 | N | N | 0 | 25 |
| 48 | 70 | M | ERM | RE | Nil | 11 | 10 | 11 | 10.667 | 9 | 11 | 7 | 9 | -1.667 | N | N | 0 | 25 |
| 49 | 74 | F | ERM | LE | Nil | 14 | 16 | 14 | 14.667 | 12 | 12 | 13 | 12.333 | -2.333 | N | N | 0 | 25 |
| 50 | 67 | F | ERM | LE | Nil | 20 | 21 | 20 | 20.333 | 21 | 19 | 20 | 20 | -0.333 | N | N | 0 | 25 |
| 51 | 71 | M | ERM | LE | Nil | 18 | 17 | 18 | 17.667 | 16 | 17 | 15 | 16 | -1.667 | N | N | 0 | 25 |
| 52 | 76 | M | ERM | RE | Nil | 18 | 18 | 17 | 17.667 | 19 | 18 | 16 | 17.667 | 0 | N | N | 0 | 25 |
| 53 | 79 | M | ERM | LE | Nil | 13 | 11 | 13 | 12.333 | 11 | 9 | 12 | 10.667 | -1.667 | N | N | 0 | 25 |
| 54 | 70 | F | ERM | RE | Nil | 15 | 16 | 16 | 15.667 | 16 | 14 | 13 | 14.333 | -1.333 | N | N | 0 | 25 |

**Supplementary Table 1: Data of all participants in Group A and Group B**

Abbreviation: M: male; F: Female; RE: Right eye; LE: Left eye; Y: Yes; N: No; TRD: Tractional retinal detachment; VH: Vitreous haemorrahge; ERM: Epiretinal membrane; SO: silicone oil; Mac: maculal; MH: macula hole; RD: retinal detachment; ROO: removal of silicone oil; G: gauge ; pIOP: Intraocular pressure at lower level; IOP: intraocular pressure at higher level
